# Beyond Bone Health: Exploring the “Heart–Brain–Bone” Axis Modulated by Lipid-Soluble Nutrients (Omega-3, Vitamin D3, and Vitamin K2)

**DOI:** 10.3390/nu18162711

**Published:** 2026-08-19

**Authors:** Shih-Chin Fang, Meng-Kai Huang, Hsieh-Tsung Ethan Shen, Bo-Xiang Benjamin Zhang, Ting-Hsuan Collette Chao, Chung-Che Wu

**Affiliations:** 1 Department of Neurology, Yonghe Cardinal Tien Hospital, New Taipei 234403, Taiwan; 2 X-Source Clinic, Taipei 115, Taiwan; 3 Master Program in School of Dental Technology, Taipei Medical University, Taipei 11031, Taiwan; 4 Sheng Hou Clinic, Taipei 10674, Taiwan; 5 YD Bio Limited, Taipei 115603, Taiwan; 6 Division of Clinical Cariology and Endodontology, Department of Oral Rehabilitation, School of Dentistry, Health Sciences University of Hokkaido, Tobetsu 061-0293, Japan; 7 Ph.D. Program in Medical Neuroscience, College of Medical Science and Technology, Taipei Medical University and National Health Research Institutes, Taipei 11031, Taiwan; 8 Department of Molecular and Cell Biology, College of Letters and Science, University of California, Berkeley, CA 94720, USA; 9 Department of Neurosurgery, Taipei Medical University Hospital, Taipei 11031, Taiwan; 10 Department of Surgery, School of Medicine, College of Medicine, Taipei Medical University, Taipei 11031, Taiwan

**Keywords:** omega-3 fatty acids, vitamin K2 (menaquinone-7), vitamin D3, matrix Gla protein, vascular calcification, specialized pro-resolving mediators, bone mineralization, inflammaging, Heart–Brain–Bone axis, narrative review

## Abstract

Background: Population aging is driving a convergent rise in three disorders historically managed in isolation: cardiovascular disease, neurocognitive decline, and osteoporotic bone loss. Mechanistic data indicate that these systems are coupled through shared regulators of calcium trafficking, inflammation resolution, vascular integrity, and inflammaging. On this basis, a “Heart–Brain–Bone” axis has been proposed; it should be understood as an integrative conceptual framework that organizes evidence drawn from three separate studies, not as a validated physiological entity with agreed diagnostic criteria or demonstrated modifiability. Three lipid-soluble nutrients—long-chain omega-3 polyunsaturated fatty acids (EPA/DHA), vitamin D3 (cholecalciferol), and vitamin K2 (menaquinone-7 [MK-7])—act on overlapping nodes of this network. Methods: We performed a structured narrative review. PubMed/MEDLINE, Embase, the Cochrane Library, and Web of Science were searched from database inception to 25 June 2026 using predefined term blocks for each nutrient, each organ domain, and each candidate mechanism, and the search was updated on 7 August 2026. Records were screened against prespecified inclusion and exclusion criteria by two authors independently, with disagreements resolved by a third. The strength of evidence for each nutrient–organ relationship was graded with an explicitly defined four-level scheme ((−) to (+++)) applied separately to preclinical, observational, randomized and meta-analytic evidence. Results: Vitamin K2-dependent gamma-carboxylation of matrix Gla protein (MGP) and osteocalcin has been proposed to influence whether calcium is incorporated into the bone matrix or deposited in the arterial wall, offering a candidate mechanistic account of the “calcium paradox” associated with isolated vitamin D3 supplementation; EPA/DHA-derived specialized pro-resolving mediators may support resolution of endothelial and neuronal inflammation; and bone-, vascular- and brain-derived signals (osteocalcin, FGF23, the neurovascular unit) interconnect the three organs. These mechanisms are biologically plausible but remain insufficiently confirmed in humans. The clinical evidence is heterogeneous, formulation- and population-dependent, and comprises positive, neutral and null results: cardiovascular omega-3 trials are discordant (REDUCE-IT, which used icosapent ethyl [an EPA ethyl ester], positive; VITAL/STRENGTH/ASCEND null, predominantly in lower-risk or replete cohorts); cognitive trials are largely null or subgroup-dependent (MAPT, DO-HEALTH, VITAL); and MK-7 improves surrogate bone and calcification biomarkers and slowed coronary artery calcification in one recent randomized imaging trial (VitaK-CAC), whereas combined MK-7 plus vitamin D3 did not slow aortic valve or coronary calcification in AVADEC and MK-7 did not reduce bone loss in early menopausal women. Recognized safety signals include a dose-dependent increase in atrial fibrillation with high-dose omega-3, adverse skeletal effects of high-dose or bolus vitamin D, and clinically relevant interference of even low-dose MK-7 with vitamin K antagonist therapy. Conclusions: No adequately powered randomized trial has demonstrated that the combination of long-chain omega-3, vitamin D3 and MK-7 is superior to its individual components or to placebo for any clinical endpoint. The combined regimen is therefore mechanistically rational and hypothesis-generating rather than clinically established; benefit appears most plausible in individuals with elevated risk or demonstrable nutritional insufficiency, and least in replete, low-risk populations. Findings should be interpreted within a broader healthy-aging context that includes lifestyle and psychosocial factors. Adequately powered factorial randomized controlled trials stratified by baseline Omega-3 index, 25(OH)D and vitamin K status, with prespecified mechanistic biomarkers and hard endpoints, are required.

## 1. Introduction

Population aging is among the most consequential epidemiological transitions of the twenty-first century, and it is accompanied by a parallel rise in chronic, degenerative, non-communicable diseases. Cardiovascular disease (CVD) remains the leading global cause of mortality; neurocognitive disorders, chief among them Alzheimer’s disease and related dementias, are projected to affect an increasing share of the population as life expectancy lengthens; and osteoporosis, with its attendant fragility fractures, imposes enormous morbidity, loss of independence, and healthcare expenditure, particularly among postmenopausal women and older men. Historically, these three disease clusters have been investigated and managed within siloed clinical disciplines—cardiology, neurology, and orthopedics/endocrinology, respectively.

This compartmentalized framework increasingly conflicts with mechanistic data demonstrating that the cardiovascular, central nervous, and skeletal systems are bound by shared regulatory pathways [1,2]. The concept of a “Heart–Brain–Bone” axis posits that these systems do not degenerate in isolation but rather decline in a coordinated, often self-reinforcing manner, governed by common upstream drivers including chronic low-grade inflammation (“inflammaging”), dysregulated calcium trafficking, endothelial dysfunction, and impaired microcirculatory perfusion [3,4]. It should be stated at the outset that this axis is a proposed integrative concept rather than an established physiological entity: it has no agreed operational definition, no diagnostic criteria, and no demonstrated modifiability as a unit. Its value is heuristic—it assembles findings that are usually reported within three separate studies into a single framework that can be interrogated—and the present review treats it accordingly throughout. The individual couplings on which the concept rests are of unequal strength. The same vascular calcification that stiffens large arteries and elevates cardiovascular risk also compromises the cerebral microvasculature that sustains neuronal metabolism, and reflects a systemic mishandling of calcium that is associated with depletion of the skeletal reservoir [1,5]. Bone is now recognized not as an inert scaffold but as an endocrine organ secreting osteocalcin and other osteokines that have been reported to exert distal effects on the vasculature and the brain, principally in experimental systems [6,7].

Within this integrative paradigm, lipid-soluble micronutrients occupy a regulatory position of interest because their receptors and molecular targets are expressed across all three organ systems. Three nutrients in particular—long-chain n-3 PUFAs (eicosapentaenoic acid, EPA; docosahexaenoic acid, DHA), vitamin D3, and vitamin K2—share a thematic mechanistic logic: each modulates inflammation, calcium handling, or membrane biophysics, and their biological actions have been proposed to be interdependent [8,9]. Vitamin D3 enhances intestinal calcium absorption; it has been hypothesized that, in the absence of adequate vitamin K2 status, a greater share of that calcium may be deposited in vascular and soft-tissue compartments rather than in bone—the proposition at the center of the “calcium paradox” [8,10]. Vitamin K2, by activating the calcium-binding Gla proteins MGP and osteocalcin, has been proposed to act as a molecular regulator that influences whether calcium is incorporated into the skeletal matrix or deposited in the arterial wall [10,11]. This “calcium-routing” formulation is a mechanistic model derived largely from in vitro work, animal models, and human surrogate-biomarker studies; it should not be read as implying that vitamin K2 physically translocates calcium from the vasculature to bone, and, as set out in Section 5 and Section 6, the clinical consequences of the model have not been confirmed. EPA and DHA, in turn, are the substrates for specialized pro-resolving mediators that actively terminate inflammation in endothelial and neural tissues [12,13].

One determinant of the plasma and tissue concentrations achievable from a given intake is the molecular form in which these nutrients are delivered. Pharmacokinetic studies indicate that re-esterified triglyceride (rTG) omega-3 concentrates may show greater bioavailability than ethyl ester (EE) forms [14,15], and that the long-chain menaquinone MK-7 has a longer circulating half-life and greater extrahepatic bioavailability than the shorter MK-4 homolog [16,17]. Such distinctions concern absorption and tissue delivery, not proven clinical superiority, and are considered in detail—together with their limits—in Section 3.

**Figure 1:**
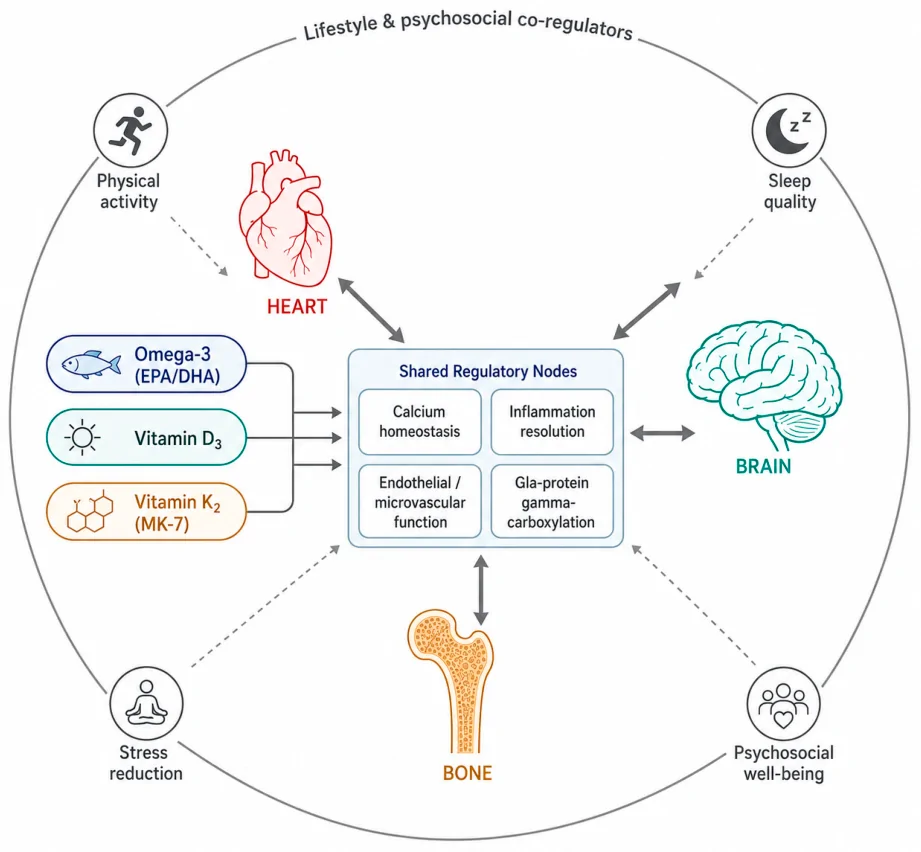
Conceptual overview of the proposed heart–brain–bone axis. Three lipid-soluble nutrients—long-chain omega-3 (EPA/DHA), vitamin D3, and vitamin K2 (MK-7)—act on shared regulatory nodes (calcium homeostasis, inflammation resolution, endothelial/microvascular function, and Gla-protein gamma-carboxylation) that have been proposed to coordinately influence cardiovascular, central nervous, and skeletal health. An outer ring depicts lifestyle and psychosocial co-regulators (physical activity, sleep quality, stress reduction, and psychosocial well-being) that modulate the same nodes, underscoring that nutrient intake is one component of a broader healthy-aging context (Section 8). The figure depicts a conceptual framework and a set of proposed relationships; it does not depict demonstrated clinical effects. Arrows and colors are used as follows: solid single-headed arrows denote the proposed input of each nutrient to the shared regulatory nodes; solid double-headed arrows denote the proposed bidirectional interaction between the shared nodes and each organ system; dashed arrows denote modulatory input from the lifestyle and psychosocial co-regulators shown in the outer ring. Colors identify elements only and encode neither the strength nor the direction of evidence: red, heart; green, brain; orange, bone and vitamin K2 (MK-7); dark blue, omega-3 (EPA/DHA); teal, vitamin D3.

The objectives of this review are fourfold. First, we set out the review methodology, including the search strategy, eligibility criteria, and the explicit framework used to grade the strength of evidence (Section 2). Second, we examine the biochemical properties and bioavailability of long-chain omega-3 (EPA/DHA), vitamin D3, and vitamin K2 (MK-7), and articulate the molecular architecture of the proposed heart–brain–bone axis—including vascular–bone coupling, neurovascular coupling, bone–brain endocrine crosstalk, and inflammaging as a common driver (Section 3 and Section 4). Third, we critically appraise the clinical trial evidence, explicitly distinguishing mechanistic, observational, and randomized data and presenting positive, neutral, and null findings alongside one another; delineate the controversies and unresolved questions that temper any therapeutic claim; and identify the populations in which supplementation is most plausibly warranted (Section 5, Section 6 and Section 7). Fourth, we situate nutrient intake within the broader lifestyle and psychosocial context that co-regulates the axis, summarize the safety signals and drug–nutrient interactions relevant to each nutrient, and describe the doses actually used in the studies reviewed (Section 8, Section 9 and Section 10). Throughout, claims are framed according to their evidentiary basis, and we deliberately avoid causal language where only mechanistic or associational data exist. Figure 1 provides a conceptual overview of the proposed axis and of the framework developed in the sections that follow.

## 2. Methods: Literature Search Strategy and Evidence Appraisal

This section reports the methodology of the review so that the evidence base and its appraisal can be reproduced and audited. It was added in response to reviewer comments on the first submission.

### 2.1. Design and Rationale

This article is a structured narrative review rather than a systematic review or a meta-analysis, and it is reported as such. It was not registered in PROSPERO and does not follow the PRISMA reporting guideline, which is designed for systematic reviews with a single, answerable clinical question. The narrative format was chosen deliberately because the subject matter spans four largely separate studies—vascular biology, neuroscience, bone metabolism, and nutritional intervention trials—that use incommensurable outcome measures and cannot be pooled quantitatively. No effect estimates were pooled, and no summary statistics were computed. To limit the selective-citation risk inherent in narrative synthesis, the search, eligibility, and appraisal steps were nevertheless prespecified and are reported in full below; the consequent methodological limitations are stated in Section 2.6.

### 2.2. Information Sources and Search Strategy

We searched PubMed/MEDLINE, Embase, the Cochrane Library (including the Cochrane Central Register of Controlled Trials), and the Web of Science Core Collection from database inception to 25 June 2026; ClinicalTrials.gov was searched for registered and ongoing trials of the three nutrients. Searches combined three term blocks—nutrient, organ/outcome, and candidate mechanism—using controlled vocabulary (MeSH/Emtree) together with free-text terms, as set out in Table 1. The search was re-executed on 7 August 2026 during preparation of this revision, both to capture studies published in the interim and to broaden the retrieval of randomized trials reporting null or neutral results; the additional trials now appraised in Section 5 were identified at that stage. The final search date for this article is therefore 7 August 2026, and this is the date reported in the Abstract and in Table 1. Searches were supplemented by backward citation tracking of the reference lists of all included trials and reviews and by forward citation tracking, and by hand-searching the tables of contents of the principal nutrition and cardiovascular journals over the final five years. Records were restricted to English-language publications.

### 2.3. Eligibility Criteria

Records were eligible if they reported (i) randomized controlled trials, or systematic reviews and meta-analyses of randomized controlled trials, of long-chain omega-3, vitamin D3, or vitamin K (including combinations) with cardiovascular, cognitive, or skeletal outcomes; (ii) prospective cohort or nested case–control studies reporting nutrient intake or status biomarkers in relation to the same outcomes; (iii) mechanistic studies in human tissue, cell or animal models that addressed a pathway explicitly linked in this review to the proposed axis; or (iv) authoritative narrative reviews, consensus statements, or dietary reference intake reports used for background and for regulatory reference values. Records were excluded if they were not peer-reviewed (preprints, conference abstracts without a full report), were case reports or case series without a mechanistic contribution, were published in a language other than English, addressed alpha-linolenic acid alone without EPA/DHA data, addressed vitamin K1 without a bearing on the menaquinone questions considered here (with the exception of the ECKO trial, retained deliberately as a null skeletal comparator), or were animal studies of pathways with no counterpart in the human evidence assembled here. Where multiple publications reported the same trial, the primary outcome publication was cited and substudies were cited only where they contributed distinct information.

### 2.4. Study Selection and Data Extraction

Titles and abstracts were screened independently by two authors (S.-C.F. and M.-K.H.) against the criteria in Section 2.3; the full texts of all potentially eligible records were then assessed by the same two authors independently. Disagreements at either stage were resolved by discussion, and residual disagreements were adjudicated by a third author (C.-C.W.). Data extracted from each included clinical study comprised design, population and sample size, intervention (including molecular form and dose), comparator, duration, prespecified primary outcome, direction of the principal result, and the limitations that bear on its interpretation; these fields populate the clinical evidence summary presented in Section 5. No formal risk-of-bias instrument (e.g., Cochrane RoB 2) was applied, consistent with the narrative design; instead, trial-level limitations material to interpretation—comparator choice, baseline nutritional status, dose, formulation, and endpoint type—are reported explicitly for every trial in that summary and are revisited in Section 6.

### 2.5. Evidence Grading Framework

Because a narrative synthesis cannot convey the strength of evidence through a pooled estimate, each nutrient–organ relationship was graded qualitatively. Four evidence streams were graded separately—preclinical, observational, randomized, and meta-analytic—using the four-level scheme defined in Table 2, and a composite “overall strength” was then assigned using the categories also defined in Table 2. Two points govern the interpretation of these gradings. First, a symbol describes the consistency and adequacy of the evidence within its own stream only: a grade of (+++) in the preclinical column denotes reproducible mechanistic demonstration across independent models and carries no implication of clinical proof. Second, the composite “overall strength” is deliberately anchored to the best available human evidence, so that a relationship supported by abundant mechanistic work but only null randomized data is graded as limited. Grading was performed independently by two authors (S.-C.F. and M.-K.H.) and reconciled by consensus, with the corresponding author adjudicating residual disagreement. This scheme is a transparent, consensus-based heuristic developed for this review; it is not a validated instrument, and it is not equivalent to GRADE, which is designed for the appraisal of a defined intervention-comparator-outcome question and cannot be applied to a mechanistic framework of this breadth. The gradings are presented in Section 5 and should be read as a structured summary of the authors’ appraisal rather than as a formal certainty rating.

### 2.6. Methodological Limitations of This Review

Four limitations of the method should be borne in mind when reading what follows. First, the review was not registered and did not apply PRISMA reporting or a formal risk-of-bias tool, so the possibility of selective citation cannot be excluded, although prespecified criteria and duplicate screening were used to constrain it. Second, the restriction to English-language publications may have excluded relevant evidence, particularly Japanese-language studies of dietary menaquinone. Third, the evidence-grading scheme, although explicitly defined, involves judgement and is not externally validated; other appraisers might grade individual cells differently, and readers are therefore directed to the underlying trials summarized in Section 5 rather than to the symbols alone. Fourth, the mechanistic literature is very much larger than can be represented in a review of this scope, and the mechanistic sections are consequently selective and illustrative rather than exhaustive.

## 3. Biochemical Properties and Bioavailability

### 3.1. Formulation and Bioavailability: rTG Versus EE and Natural TG Forms of Omega-3

Marine omega-3 fatty acids are available in several molecular configurations whose digestion and absorption differ substantially. In native fish, EPA and DHA exist predominantly as natural triglycerides (TG). During industrial concentration, the triglyceride is transesterified to the ethyl ester (EE) form, yielding a high-purity concentrate in which each fatty acid is bound to an ethanol moiety. Enzymatic re-esterification of the EE concentrate back onto a glycerol backbone produces the re-esterified triglyceride (rTG) form, combining high purity with a near-native structure [15].

The pharmacological relevance derives from lipid-digestion mechanics. Pancreatic lipase exhibits higher catalytic efficiency toward the glycerol-esterified sn-1 and sn-3 positions of triglycerides than toward the ethyl ester bond; EE forms are therefore hydrolyzed more slowly and depend more heavily on co-ingested dietary fat [15]. A controlled bioavailability study reported that the rTG form achieved higher EPA + DHA bioavailability (approximately 124% relative to natural fish oil) than the EE form (approximately 73%) [14]. Because steady-state erythrocyte membrane EPA/DHA content (the Omega-3 index) governs eicosanoid and pro-resolving mediator synthesis, differences in absorption may translate into differences in the substrate available for downstream signaling [12,14], an effect that would be expected to be most relevant in older or low-fat-diet populations with attenuated digestive lipase activity; this expectation is itself untested. Two caveats are essential. First, this evidence derives from pharmacokinetic (human bioavailability) studies and short-term biomarker endpoints, not clinical outcomes. Although rTG formulations may show superior bioavailability compared with ethyl ester forms in pharmacokinetic studies, no adequately powered randomized trial has demonstrated superior cardiovascular, cognitive, or skeletal clinical outcomes attributable to the rTG formulation. Second, bioavailability is not equivalent to clinical efficacy: the trials that have shaped the omega-3 outcome literature (Section 5) have almost exclusively used ethyl ester or carboxylic-acid preparations rather than rTG, so formulation and clinical outcome cannot presently be equated. For these reasons, the remainder of this review treats long-chain omega-3 (EPA/DHA) as the entity of interest, and confines formulation-specific claims to bioavailability.

### 3.2. Vitamin D3 (Cholecalciferol): Activation and Receptor Biology

Vitamin D3 is a secosteroid pro-hormone, biologically inert until sequentially hydroxylated: hepatic 25-hydroxylase (CYP2R1) generates 25-hydroxyvitamin D [25(OH)D], the principal circulating storage metabolite and status biomarker, which renal 1-alpha-hydroxylase (CYP27B1) converts to the active 1,25-dihydroxyvitamin D (calcitriol) [18,19]. Calcitriol acts through the nuclear vitamin D receptor (VDR), a ligand-activated transcription factor that heterodimerizes with the retinoid X receptor (RXR) and binds vitamin D response elements in target gene promoters [19]. VDR is expressed not only in the intestines, the kidney, and bones but also in cardiomyocytes, vascular smooth muscle, neurons, and glia, providing a molecular basis for the pleiotropic actions attributed to vitamin D3 across the axis [19,20]. As a lipophilic secosteroid, vitamin D3 absorption is enhanced by co-administration with dietary lipids, which mechanistically complements a triglyceride-based omega-3 matrix. Because vitamin D3 increases the systemic calcium load, its supplementation has been proposed to heighten the requirement for the calcium-handling machinery discussed in Section 4; whether this translates into a clinically relevant requirement for concomitant vitamin K is examined in Section 4.1, Section 5.1 and Section 6.

### 3.3. Vitamin K2 Pharmacokinetics: MK-7 Versus MK-4

Vitamin K2 comprises menaquinones (MK-n) distinguished by the number (n) of isoprenoid side-chain units. MK-4 is rapidly absorbed but has a very short plasma half-life (1–2 h), so physiologically relevant systemic concentrations require high, divided doses [16]. MK-7, by contrast, has a longer side chain conferring greater lipoprotein binding, a circulating half-life measured in days, and greater accumulation in extrahepatic tissues such as the arterial wall and bone [16,17]. This extended residence time is functionally important: sustained plasma MK-7 provides continuous delivery of the cofactor to gamma-glutamyl carboxylase in extrahepatic tissues, supporting ongoing carboxylation—and hence activation—of MGP and osteocalcin between doses [10,17]. A once-daily MK-7 regimen of approximately 90–180 micrograms maintains Gla-protein carboxylation more effectively than equivalent or higher doses of short-acting MK-4 [16,21]. It should be noted that the endpoint in these comparisons is the carboxylation status of circulating Gla proteins, a surrogate marker; no head-to-head trial has compared MK-7 with MK-4 for a clinical endpoint. The comparative biochemical and pharmacokinetic properties of the three nutrients are summarized in Table 3.

## 4. The Proposed “Heart–Brain–Bone” Axis: Molecular Mechanisms and Potential Complementarity

The central proposition examined in this review is that the three nutrients may act not as independent agents but as a functionally integrated module. The following subsections first dissect organ-specific mechanisms (Section 4.1, Section 4.2 and Section 4.3) and then develop the inter-organ coupling that constitutes the proposed axis (Section 4.4, Section 4.5, Section 4.6 and Section 4.7), before synthesizing the integrated crosstalk (Section 4.8); the mechanistic roles are consolidated in Table 4. The whole of this section describes mechanisms and their supporting preclinical evidence; the human clinical evidence that tests them is appraised separately in Section 5, and the two should not be conflated.

### 4.1. Cardiovascular Protection and the Calcium Paradox

The “calcium paradox” describes the observation that calcium essential for skeletal integrity becomes pathogenic when deposited ectopically in the arterial wall. Vitamin D3, by enhancing intestinal calcium absorption, increases the bioavailable calcium pool; it has been proposed that in subclinical vitamin K2 deficiency, a greater fraction of this calcium may be deposited in the vascular intima and media rather than in bone [8,10]. The principal endogenous inhibitor of this deposition is matrix Gla protein (MGP), synthesized by vascular smooth muscle cells [10]. MGP is functional only after vitamin K-dependent carboxylation: gamma-glutamyl carboxylase, using MK-7 as a cofactor, converts specific glutamate residues to gamma-carboxyglutamate (Gla) residues [10,11]. Carboxylated MGP binds calcium and hydroxyapatite and sequesters bone morphogenetic protein-2, preventing osteogenic transdifferentiation of vascular smooth muscle cells [5,10]. In vitamin K2 insufficiency, MGP remains undercarboxylated (ucMGP) and loses protective capacity; circulating dephospho-uncarboxylated MGP is an established biomarker of vascular calcification risk [22,23]. It has therefore been hypothesized that vitamin K status may modify the vascular handling of the additional calcium made available by vitamin D3 [8,9]. This proposition is biologically plausible but remains insufficiently confirmed in humans, and three qualifications are necessary. First, the evidence that carboxylation status determines the site of calcium deposition is derived from in vitro systems, from Mgp-null animals, and from human studies in which dephospho-uncarboxylated MGP served as the outcome; it does not establish translocation of calcium between compartments. Second, the claim sometimes made in the wider literature that vitamin K2 is required for the safe use of vitamin D3 is not supported by current human evidence and is not made here. Third, and most directly, the AVADEC randomized trial found that two years of combined MK-7 (720 micrograms/day) and vitamin D (25 micrograms/day) in 365 elderly men did not slow aortic valve calcification, and that progression of aortic and coronary artery calcification scores did not differ significantly between groups, despite a marked reduction in dephospho-uncarboxylated MGP [24]—a clear demonstration that modification of the biomarker does not guarantee modification of the calcification process.

**Figure 2:**
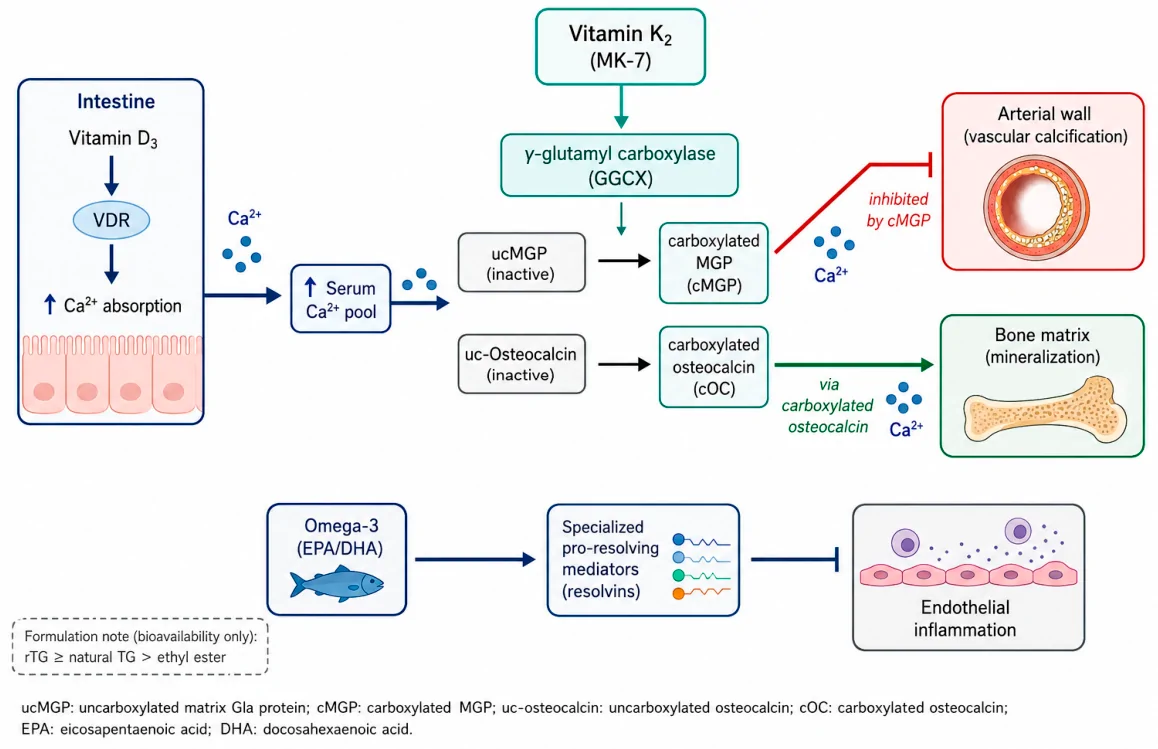
Proposed molecular account of the calcium paradox. Vitamin D3 increases intestinal calcium absorption; vitamin K2 (MK-7) supplies gamma-glutamyl carboxylase with the cofactor required to carboxylate matrix Gla protein (MGP) and osteocalcin, which is proposed to favor incorporation of calcium into the bone matrix (via carboxylated osteocalcin) and to inhibit mineralization of the arterial wall (via carboxylated MGP). EPA/DHA-derived specialized pro-resolving mediators concurrently attenuate endothelial inflammation. The scheme depicts a mechanistic hypothesis supported principally by in vitro, animal, and surrogate-biomarker data; it does not depict a demonstrated translocation of calcium between compartments, and the clinical consequences of the model have not been confirmed in adequately powered randomized trials (Section 5.1 and Section 6).

Acting in parallel, long-chain n-3 PUFAs may confer complementary protection through active resolution of inflammation. Once incorporated into endothelial and leukocyte membranes, EPA and DHA serve as substrates for specialized pro-resolving mediators (SPMs)—resolvins, protectins, and maresins—generated via lipoxygenase and cyclooxygenase-2 pathways [12,13]. Unlike passive dilution of arachidonic acid, SPMs are agonists that actively terminate inflammation: they limit neutrophil infiltration, promote macrophage efferocytosis, and downregulate NF-kappaB-driven adhesion molecules (VCAM-1, ICAM-1) and cytokines (TNF-alpha, IL-6) [12,13]. By attenuating endothelial dysfunction and lowering triglycerides, omega-3 addresses an inflammatory dimension of cardiovascular risk that calcium handling alone does not [13,25]. At the mechanistic level, these actions may be complementary: K2-dependent carboxylation is proposed to influence where calcium is deposited, whereas omega-3-derived mediators are proposed to modulate the inflammatory milieu associated with vascular injury. Both propositions are biologically plausible but insufficiently confirmed in humans; the corresponding clinical outcome evidence, which is discordant, is appraised in Section 5.1. Figure 2 summarizes this proposed molecular account of the calcium paradox and the parallel resolution pathway.

### 4.2. Neuroprotection and Cognitive Mechanisms

The brain is exceptionally lipid-rich, and DHA is its most abundant long-chain PUFA, a major structural component of neuronal and synaptic membrane phospholipids. DHA enrichment modulates membrane fluidity, the function of integral membrane proteins and ion channels, and synaptic vesicle trafficking, supporting synaptic plasticity and neurotransmission [26]. DHA is also the precursor of neuroprotectin D1 (NPD1), an SPM that suppresses neuronal apoptosis, attenuates microglial neuroinflammation, and promotes non-amyloidogenic processing of amyloid precursor protein in preclinical models [26,27]. Vitamin D3 exerts independent neuroprotective effects via CNS-expressed VDR: calcitriol regulates neurotrophic factor transcription, limits excitotoxic calcium influx by downregulating L-type voltage-gated calcium channels, and supports amyloid-beta clearance in experimental systems [20]. Vitamin K contributes through sphingolipid metabolism—serving as a cofactor in the synthesis of brain-enriched sphingolipids—and through carboxylation of the Gla protein Gas6, a ligand for TAM-family receptor tyrosine kinases that promotes neuronal survival and oligodendrocyte myelination [28,29]. Causal animal evidence links warfarin-induced brain vitamin K deficiency to altered sphingolipid profiles and cognitive-behavioral deficits [30]. Each of these mechanisms is biologically plausible but remains insufficiently confirmed in humans: the human cognitive trial data appraised in Section 5.2 are substantially less consistent than these mechanistic and animal findings, and no human study has shown that any of the pathways described here mediate a cognitive effect of supplementation.

### 4.3. Bone Remodeling and Mineralization

**Figure 3:**
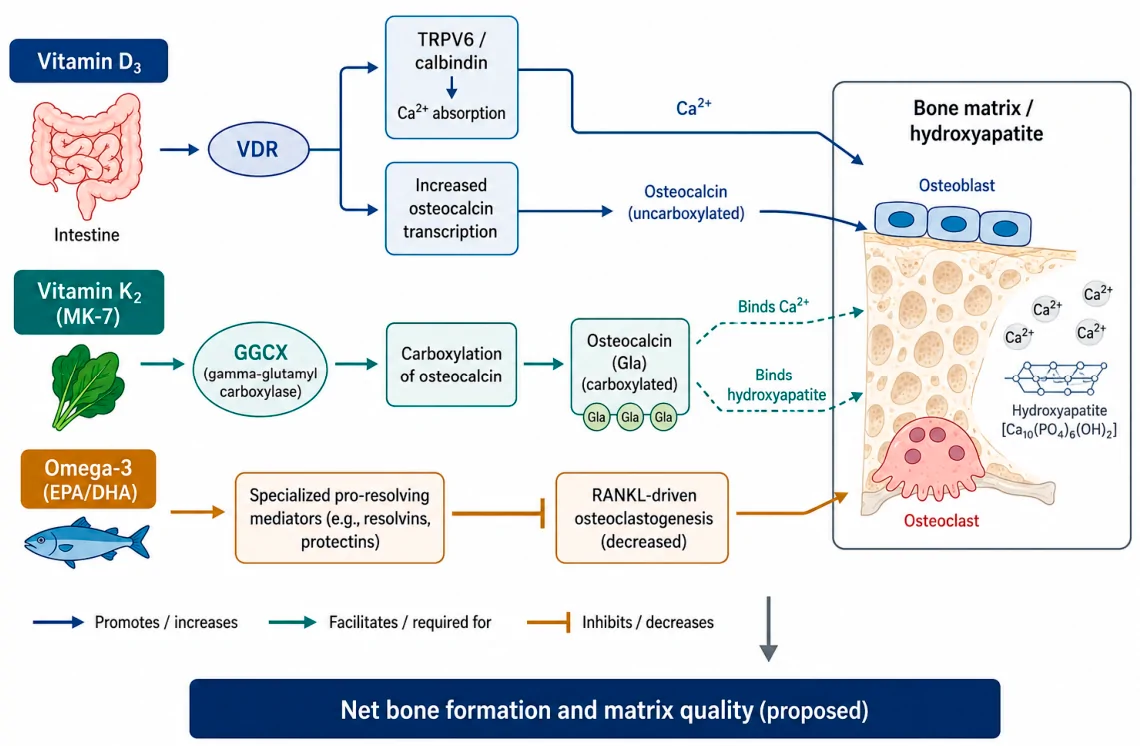
Proposed integrated mechanisms of omega-3, vitamin D3, and MK-7 in bone mineralization. Vitamin D3 secures calcium supply (TRPV6/calbindin) and upregulates osteocalcin transcription; vitamin K2 (MK-7) carboxylates osteocalcin to its high-affinity form; omega-3 PUFAs dampen RANKL-driven osteoclastogenesis—together proposed to favor net bone formation and matrix quality. The scheme summarizes mechanistic evidence; the corresponding randomized clinical evidence is limited to biomarker and bone-density endpoints and is appraised in Section 5.3.

Skeletal homeostasis depends on coupling osteoclast-mediated resorption with osteoblast-mediated formation. Vitamin D3, via calcitriol-VDR signaling in the intestinal epithelium, upregulates the apical calcium channel TRPV6 and cytosolic calbindin-D9k, driving active transcellular calcium absorption and maintaining the calcium-phosphate supersaturation required for mineralization [18,19]. Vitamin K2 governs the qualitative incorporation of that calcium by activating osteocalcin (bone Gla protein), the most abundant non-collagenous bone protein [11,21]. Newly synthesized osteocalcin is undercarboxylated with low hydroxyapatite affinity; gamma-glutamyl carboxylase, using MK-7, carboxylates three glutamate residues to generate carboxylated osteocalcin, which anchors mineral to the collagenous matrix [11,31]. The proportion of undercarboxylated osteocalcin is an inverse marker of vitamin K status associated in cohorts with lower bone mineral density [21,31]. The proposed D3–K2 relationship in bone mirrors that advanced for the vasculature: D3 controls calcium availability and upregulates osteocalcin transcription, while K2 is required for the functional activity of the protein [8]. n-3 PUFAs may further support bone indirectly by attenuating the pro-inflammatory cytokines that drive RANKL-mediated osteoclastogenesis [13]. Bone is the organ for which this mechanistic account is best matched by human data, but even here the randomized evidence is confined to biomarker and bone-density endpoints and is not uniformly positive (Section 5.3). These proposed mechanisms are summarized in Figure 3.

### 4.4. Vascular–Bone Coupling

The best-supported pillar of the proposed axis is the bone–vascular coupling captured by the calcification paradox: epidemiologically, low bone mineral density and arterial calcification co-occur more often than chance, and progress in parallel with age, independent of conventional risk factors [1,2]. This inverse bone–vessel relationship does not appear to be merely a coincidence; it has been attributed to a shared set of molecular regulators. Vascular calcification is an active, cell-mediated process in which contractile vascular smooth muscle cells transdifferentiate into an osteoblast-like phenotype, upregulating the master osteogenic transcription factor RUNX2 and depositing a mineralized matrix recapitulating bone formation [5]. The same signaling families that govern skeletal remodeling—the RANK/RANKL/osteoprotegerin (OPG) triad, bone morphogenetic proteins, and Wnt/beta-catenin-operate in the vessel wall, such that dysregulation may simultaneously promote bone resorption and arterial mineralization [1,32]. MGP sits at the center of this proposed coupling: as a vitamin K-dependent inhibitor expressed in both compartments, its carboxylation status is proposed to determine whether calcium and BMP-2 signaling drive physiological bone mineralization or pathological vascular calcification [10,32]. The bone–vascular axis is best characterized in chronic kidney disease, where mineral-bone disorder and accelerated vascular calcification are tightly linked, and the principle has been extrapolated to vascular aging [32]. This subsection rests primarily on observational and mechanistic evidence; intervention data confirming that modulating the shared pathways alters both endpoints concurrently remain absent.

### 4.5. Neurovascular Coupling and Blood–Brain Barrier Integrity

The brain’s dependence on its microcirculation provides the second proposed pillar. The neurovascular unit—the functional ensemble of endothelial cells, pericytes, astrocytic end-feet, and neurons—matches regional cerebral blood flow to metabolic demand and maintains the blood–brain barrier (BBB) [4]. Endothelial dysfunction, arterial stiffness, and microvascular calcification impair neurovascular coupling and BBB integrity, producing chronic cerebral hypoperfusion and the small-vessel pathology that underlies vascular cognitive impairment [4,33]. With aging, BBB breakdown permits extravasation of neurotoxic plasma components, perivascular inflammation, and impaired clearance of amyloid-beta mechanisms increasingly implicated in neurodegeneration [33,34]. Because the nutrient triad acts on this vascular substrate—omega-3-derived SPMs preserving endothelial function and resolving perivascular inflammation, vitamin K2-activated MGP proposed to limit cerebral microvascular calcification, and vitamin D3 supporting endothelial and BBB-stabilizing signaling [13,20,35]—it has been argued that cardiovascular protection should extend mechanistically to the maintenance of cerebral perfusion. A recent review develops this vitamin K2-vascular-cognition route explicitly [35]. The route is biologically plausible but remains insufficiently confirmed in humans: no trial has demonstrated that any of these nutrients improves neurovascular coupling, cerebral perfusion, or BBB integrity in people, and the cognitive trial evidence is largely null (Section 5.2).

### 4.6. Bone–Brain and Bone–Heart Endocrine Crosstalk

The third proposed pillar reframes bone as an endocrine organ whose secreted factors (osteokines) act on distant targets [6,36]. The vitamin K-dependent Gla protein osteocalcin is the prototypical example: beyond anchoring bone mineral, its undercarboxylated fraction enters the circulation as a hormone. In rodent models, osteocalcin crosses the blood–brain barrier, binds neurons, promotes monoamine neurotransmitter synthesis, and acts in the hippocampus via the receptor GPR158 to enhance learning and memory and counter age-related cognitive decline [7,37]. The same Gla-protein carboxylation machinery that activates osteocalcin for bone is therefore positioned, in principle, to influence its endocrine signaling to the brain, offering a vitamin K2-centered mechanistic thread linking the skeletal and neural poles of the proposed axis [7,38]. Bone also signals to the heart: the osteocyte-derived phosphaturic hormone FGF23, elevated in mineral-bone disorders, directly induces left-ventricular hypertrophy via calcineurin-NFAT signaling, connecting skeletal mineral metabolism to cardiac remodeling [39]. Osteokines including osteocalcin, FGF23, and osteoprotegerin have additionally been implicated in atherosclerosis [36,40]. This pillar is the weakest of the three: the bone–brain and bone–heart links derive almost entirely from preclinical and mechanistic studies, the osteocalcin-GPR158 pathway has not been demonstrated in humans, and the human relevance of osteocalcin’s neuroendocrine actions is unresolved. These mechanisms are biologically plausible but remain insufficiently confirmed in humans, and no nutritional intervention has been shown to modify them clinically.

### 4.7. Inflammaging as a Common Upstream Driver

Underlying all three proposed pillars is inflammaging—the chronic, sterile, low-grade inflammation that accompanies aging and is now recognized as a hallmark of the aging process [3,41]. Inflammaging is sustained by immunosenescence, accumulation of senescent cells secreting a pro-inflammatory secretome, oxidative stress, and mitochondrial dysfunction, and it operates in a self-amplifying loop with the other hallmarks of aging [42,43,44]. This shared inflammatory substrate offers a unifying explanation for the co-segregation of cardiovascular, cognitive, and skeletal decline: persistent NF-kappaB activation simultaneously promotes endothelial dysfunction, neuroinflammation, and RANKL-driven bone resorption [42,45]. The nutrient triad intersects this driver at multiple points—EPA/DHA-derived SPMs actively resolve inflammation rather than merely suppressing it [12], vitamin D3 exerts anti-inflammatory transcriptional effects via VDR [19], and vitamin K has been reported to attenuate pro-inflammatory signaling [11]. By dampening a common upstream amplifier, the components of the triad could in principle exert correlated benefit across the axis. This too is biologically plausible but insufficiently confirmed in humans; indeed, the AVADEC substudy of combined vitamin K2 and D3 reported no effect on epicardial adipose tissue or on systemic inflammatory markers, illustrating that an anti-inflammatory action cannot be assumed from mechanism alone [46].

### 4.8. Integrated Crosstalk: A Unifying Synthesis

Synthesizing Section 4.4, Section 4.5, Section 4.6 and Section 4.7, the proposed heart–brain–bone axis can be understood as a network with two shared currencies—calcium and inflammation—propagated along a common vascular conduit and modulated by endocrine osteokine signaling. Misrouted calcium is proposed to link bone loss to vascular and cerebral microvascular calcification (vascular–bone and neurovascular coupling); unresolved inflammaging concurrently degrades endothelium, neurons, and bone; and bone-derived hormones are proposed to feed back onto heart and brain. The three nutrients map onto this network with complementary, non-redundant roles, summarized in Table 4: vitamin K2 is implicated in calcium handling and Gla-protein-mediated osteokine activation; vitamin D3 in calcium supply and VDR-mediated pleiotropy across all three organs; and long-chain omega-3 (EPA/DHA) in the inflammatory milieu and membrane lipid composition of vasculature and brain. The mechanistic case for complementarity is internally coherent; it is, however, a hypothesis, and the remainder of this review tests it against clinical evidence that is considerably more equivocal.

## 5. Clinical Evidence: Trials of the Individual Nutrients

Mechanistic plausibility must be distinguished from demonstrated clinical benefit. This section appraises the human evidence by organ system and study design. Positive, neutral, and null results are reported alongside one another, and trials whose results do not support the mechanistic account developed in Section 4 are given the same prominence as those that do. The overall strength of evidence by topic is graded in Table 5, using the framework defined in Section 2.5 and Table 2; the major clinical trials, including the direction of their principal result, are summarized in Table 6.

### 5.1. Cardiovascular Outcomes

The omega-3 cardiovascular literature is the most extensive and the most discordant. The GISSI-Prevenzione trial (*n* = 11,324 post-myocardial-infarction patients, ~1 g/day n-3 PUFA) reported reduced death and cardiovascular events, providing early secondary-prevention support [47]. Decades later, REDUCE-IT (*n* = 8179 statin-treated patients with elevated triglycerides) found that high-dose (4 g/day) icosapent ethyl-a purified EPA ethyl ester, and specifically not an rTG preparation—reduced major adverse cardiovascular events by approximately 25% [48], and the EVAPORATE imaging substudy demonstrated regression of low-attenuation coronary plaque on serial CT [49]. It is therefore notable that the single most influential positive cardiovascular omega-3 trial used an ethyl ester formulation, so this signal provides no support for any claim of rTG clinical superiority. In stark contrast, three large randomized trials were null: VITAL (*n* = 25,871, 1 g/day EPA + DHA, primary prevention), ASCEND (*n* = 15,480, diabetes), and STRENGTH (*n* = 13,078, 4 g/day mixed EPA + DHA carboxylic acid versus corn oil) all failed to reduce major cardiovascular events [50,51,52]. The divergence between REDUCE-IT and STRENGTH—both at high doses—has fueled debate over EPA-only versus EPA + DHA formulations and the choice of mineral-oil versus corn-oil comparators (Section 6). Recent meta-analyses partially reconcile these data: pooled analyses report modest reductions in cardiovascular events that are larger for EPA-only trials and for higher doses, and an inverse association of EPA + DHA with sudden cardiac death, alongside a dose-dependent atrial-fibrillation safety signal [53,54,55,56] (Section 9.1). For vitamin K2, the evidence had until recently been confined to observational data (the Rotterdam Study associated higher dietary menaquinone intake with lower coronary heart disease and aortic calcification) and surrogate-biomarker or intermediate-endpoint randomized trials, of which the most informative reported improved carotid and aortic stiffness indices after three years of MK-7 180 micrograms/day in 244 healthy postmenopausal women [57,58]. The randomized picture that has since emerged is mixed rather than uniformly favorable. VitaK-CAC, a two-year trial in 180 patients with coronary artery disease, reported significantly less progression of the coronary artery calcification score with MK-7 360 micrograms/day than with placebo [59,60], and remains the only randomized trial to show an effect of a menaquinone on a hard imaging endpoint. Against this, AVADEC randomized 365 elderly men with aortic valve calcification to MK-7 720 micrograms/day plus vitamin D 25 micrograms/day or placebo for two years and found no effect on aortic valve calcification progression, no significant difference in aortic or coronary calcification score progression, and no difference in valve surgery, death or cardiovascular events, despite a large fall in dephospho-uncarboxylated MGP [24]; its substudy likewise modified the vitamin K-status marker without significantly changing epicardial adipose tissue or systemic inflammation [46]. A further randomized trial in 68 patients with type 2 diabetes found that six months of MK-7 360 micrograms/day reduced dephospho-uncarboxylated MGP but did not reduce arterial calcification on computed tomography [61]. Taken together, the vascular evidence for vitamin K2 shows a consistent and large effect on the carboxylation biomarker with an inconsistent and, at best, modest effect on imaging endpoints; this dissociation is central to the interpretation offered in Section 6.

### 5.2. Cognitive Outcomes

Cognitive trial evidence is weaker and predominantly null at the level of confirmatory randomized trials. The MIDAS trial (*n* = 485, 900 mg/day DHA, 24 weeks) reported improved episodic and visuospatial memory in healthy older adults with age-related cognitive decline [62]. However, the larger and longer MAPT trial (*n* = 1680, omega-3 with or without multidomain intervention, 3 years) found no significant overall cognitive benefit, with a signal only in pre-specified high-risk subgroups [63], and the factorial DO-HEALTH trial (*n* = 2157; vitamin D3, omega-3, and exercise in generally healthy seniors) found no effect on its prespecified clinical outcomes [64]. For vitamin D specifically, the VITAL cognitive ancillary studies (*n* = 3424 assessed by telephone and *n* = 794 in person) found no effect of 2000 IU/day cholecalciferol on the rate of decline in a global cognitive composite, and no interaction with the baseline 25(OH)D concentration [65]. Observational evidence is more favorable: a large pooled analysis (>100,000 participants) associated higher omega-3 intake, supplementation, and blood levels with lower dementia and cognitive-decline risk, with the strongest signal in APOE epsilon-4 carriers [66], and higher omega-3 status has been linked to better memory and greater regional brain volume [67]. Meta-analyses likewise associate low 25(OH)D with higher odds of Alzheimer’s disease and a dose-dependent increase in dementia risk [68,69], and a recent review links vitamin K2 to cognition through the vascular route [35]; no randomized trial of vitamin K and cognition with a clinical endpoint has been reported. The recurring pattern—positive observational associations not confirmed by randomized trials—is the central interpretive challenge (Section 6), and on current evidence the cognitive arm of the proposed axis is the least supported clinically.

### 5.3. Bone Outcomes

Bone evidence is the most consistent of the three domains, but it too is confined to surrogate and bone-density endpoints and is not uniform. The pivotal Knapen trial (*n* = 244 postmenopausal women, MK-7 180 micrograms/day, 3 years) demonstrated improved osteocalcin carboxylation and significantly reduced age-related bone mineral density loss at the lumbar spine and femoral neck [21]. Recent meta-analyses corroborate that K2 supplementation raises carboxylated osteocalcin and lowers undercarboxylated osteocalcin in postmenopausal osteoporosis, and that habitual dietary MK-7 (natto) intake is associated with osteocalcin carboxylation and bone density [31,70]. A 2025 prospective study reported that combined K2 + D3 improved spinal fusion outcomes in osteoporotic patients [71]. Two randomized results temper this picture. In early menopausal women (*n* = 334), 12 months of MK-7 delivered as natto reduced undercarboxylated osteocalcin but did not influence bone loss at the total hip or any other site [72], and in the ECKO trial, 5 mg/day of vitamin K1 for two to four years in postmenopausal women with osteopenia did not protect against the age-related decline in bone mineral density, although fewer fractures and cancers were observed in exploratory analyses [73]. The carboxylation biomarker therefore responds reliably to vitamin K while the density response does not, mirroring the dissociation seen in the vascular literature. For vitamin D3, the role in calcium absorption and musculoskeletal maintenance is well established mechanistically, but the VITAL fracture ancillary study—the largest randomized test to date (*n* = 25,871, 2000 IU/day, median 5.3 years)—found no reduction in total, non-vertebral or hip fractures in a generally healthy population not selected for deficiency or low bone mass [74]. Overall, bone-density and biomarker endpoints constitute the strongest clinical pillar for the triad, but no randomized trial has tested the combination against fracture incidence.

### 5.4. Balance of Positive, Neutral and Null Findings

It is worth stating plainly how the trial evidence distributes across the three domains because the mechanistic sections above could otherwise convey a more favorable impression than the data support. Of the nineteen studies summarized in Table 6, seven reported a positive result on their principal endpoint—GISSI-Prevenzione and REDUCE-IT on cardiovascular events, MIDAS on memory tests, EVAPORATE and VitaK-CAC on imaging endpoints, and the two Knapen trials on bone mineral density and arterial stiffness respectively—whereas twelve were null or neutral: STRENGTH, VITAL, ASCEND, the VITAL fracture and cognitive ancillary studies, MAPT, DO-HEALTH, AVADEC and its substudy, the diabetes calcification trial, the early-menopausal MK-7 trial, and ECKO. Within the null group, a distinct subset—AVADEC and its substudy, the diabetes calcification trial, and the early-menopausal MK-7 trial—demonstrated the expected vitamin K biomarker response without any corresponding clinical or structural benefit. Among the cardiovascular omega-3 trials the positive results cluster in selected higher-risk populations given high doses, and the null results in replete or lower-risk cohorts given lower doses; that pattern is the empirical basis for the population-dependence argument developed in Section 7, but it is a post hoc reading of heterogeneous trials rather than a tested hypothesis, and it should not be mistaken for evidence of efficacy in the populations concerned. The vitamin K trials do not follow the same pattern, since the highest dose tested produced the clearest null result (AVADEC). Two asymmetries deserve particular emphasis. First, every trial that has measured a vitamin K-status biomarker has shown it to respond, whereas the structural and clinical endpoints have responded inconsistently; a biomarker response is therefore a poor proxy for benefit here. Second, and most importantly, no trial in Table 6 tested the three nutrients together against their individual components, so the central proposition of this review has not been examined experimentally at all.

## 6. Limitations, Controversies, and Unresolved Questions

A balanced appraisal must foreground the substantial uncertainties that qualify the preceding mechanistic narrative.

Omega-3: The discordance among high-quality cardiovascular randomized trials is unresolved. The opposing results of REDUCE-IT (positive) and STRENGTH (null) have been attributed variously to the EPA-only versus EPA + DHA composition, dose and achieved plasma EPA levels, and—controversially—to possible harm from the mineral-oil placebo used in REDUCE-IT, raising the comparator’s event rate [48,52]. Null primary-prevention trials (VITAL, ASCEND) used lower (1 g/day) doses, complicating cross-trial comparison [50,51]. A dose-dependent increase in atrial fibrillation is a consistent safety signal across high-dose trials and meta-analyses [55,56,75] and is examined in Section 9.1. Formulation heterogeneity (rTG vs. EE vs. carboxylic acid) further confounds the literature [15]. Two interpretive points follow. First, greater bioavailability is not equivalent to greater clinical efficacy: a formulation may raise the Omega-3 index more efficiently without any demonstrated advantage in cardiovascular, cognitive, or skeletal endpoints, and no trial has isolated formulation as the determinant of outcome. Second, the totality of evidence argues against describing omega-3 as uniformly effective in the general population; it is more accurately characterized as a population- and baseline-status-dependent intervention whose benefit, where present, concentrates in higher-risk or nutritionally insufficient individuals (Section 7) [48,50].

Vitamin D: Cognitive and many extraskeletal outcomes are inconsistent, and the largest randomized tests have been null: neither the VITAL fracture ancillary study nor the VITAL cognitive ancillary studies found benefit in a generally healthy, largely replete population [65,74]. A recurring theme is baseline-status dependency: benefit, where seen, concentrates in deficient individuals, whereas supplementation of replete populations yields little (the basis for several null trials such as DO-HEALTH) [19,64]. This interpretation is, however, itself incompletely tested, since few trials have enrolled exclusively deficient participants. The shape of the dose–response relationship and the optimal target 25(OH)D concentration remain contested; higher is demonstrably not better, since high daily doses have been associated with lower volumetric bone density and bolus regimens with increased falls and fractures (Section 9.2). Most positive cognitive data are observational and vulnerable to reverse causation and confounding [68,69].

Vitamin K2: The principal limitation is the reliance on surrogate biomarkers—undercarboxylated osteocalcin, dephospho-uncarboxylated MGP, bone mineral density, coronary calcium score—rather than hard clinical endpoints [21,22]. The evidence accumulated since 2019 has sharpened rather than resolved the problem: MK-7 reliably and substantially lowers dephospho-uncarboxylated MGP in every trial that has measured it, yet the downstream structural endpoints have moved in one trial (VitaK-CAC) and not in others (AVADEC, the diabetes calcification trial), and the skeletal density endpoint moved in one postmenopausal trial (Knapen) and not in the other (Emaus) [24,59,61,72]. Two readings are possible—that the populations, doses, and durations differed materially, or that the carboxylation biomarker is not on the causal path to the clinical outcome—and the available data do not discriminate between them. Large fracture- and cardiovascular-event randomized trials are absent; VitaK-CAC is an important advance but reports an imaging endpoint in 180 patients rather than clinical events, and requires replication [59]. Optimal dose, the MK-7 versus MK-4 question in hard-endpoint terms, and the interaction with vitamin K antagonists (Section 9.3) all remain open.

The heart–brain–bone axis: Most fundamentally, the mechanistic evidence for the axis substantially exceeds the clinical evidence for coordinated, modifiable benefit, and the axis remains a proposed organizing concept rather than a demonstrated, modifiable physiological unit. The bone–vascular coupling is well supported observationally and mechanistically, but bone–brain endocrine signaling (e.g., osteocalcin-GPR158) rests largely on rodent models of uncertain human relevance [7,37]. It must be stated explicitly, and it is the single most important limitation of this review: no adequately powered randomized controlled trial has demonstrated that the combined intervention—long-chain omega-3 together with vitamin D3 and MK-7—is superior to its individual components, or to placebo, for any cardiovascular, cognitive, or skeletal clinical endpoint. Indeed, no trial has tested the full triad against its components at all; the only randomized data on any pairwise combination come from AVADEC, in which vitamin K2 plus vitamin D3 did not slow valvular or arterial calcification [24], and from DO-HEALTH, in which vitamin D3 plus omega-3 (with or without exercise) did not improve the prespecified clinical outcomes [64]. The complementarity hypothesis is therefore mechanistically motivated and clinically unproven, and the two randomized tests of pairwise combinations that do exist were both null. Causality—as opposed to association—remains to be established for most of the axis-level claims, and nothing in this review should be read as a recommendation to use the combination in clinical practice.

## 7. Who May Benefit Most from Supplementation?

The heterogeneity of omega-3 trial results (Section 5.1 and Section 6) is increasingly interpreted not as evidence of no effect, but as evidence that any effect is conditional on the population and its baseline status. Framing omega-3 as universally beneficial is not supported by the randomized evidence; a more defensible position is that omega-3 is a population- and baseline-status-dependent intervention, most plausibly warranted in the following, overlapping groups. It should be borne in mind that this stratification is derived post hoc from between-trial comparisons and subgroup analyses; with the partial exception of hypertriglyceridaemia, none of these groups has been prospectively validated as a target for supplementation.

Hypertriglyceridemia and elevated cardiometabolic risk: The most consistent randomized signal arises in statin-treated patients with elevated triglycerides, in whom high-dose EPA (as icosapent ethyl) reduced cardiovascular events [46]. High-dose omega-3 also lowers triglycerides and may modify other cardiometabolic risk factors [24].

Secondary prevention and established high cardiovascular risk: Early secondary-prevention data in post-myocardial-infarction patients suggested benefit [45], and recent meta-analyses report that event reductions are larger in higher-risk populations and with higher-dose, EPA-predominant regimens [52,54]. By contrast, primary-prevention trials in generally healthy or lower-risk cohorts (VITAL, ASCEND) were null [48,49].

Low baseline omega-3 status or low habitual fish intake: Benefit is mechanistically more plausible where the baseline Omega-3 index is low, and there is headroom for membrane enrichment; older adults with low habitual marine intake are a pertinent example. Several null trials enrolled cohorts that were relatively replete or at low risk, which may have limited their capacity to detect benefit [48,54,61].

Cognitive-risk subgroups (interpreted with caution): Observational data associate higher omega-3 intake and status with lower dementia risk, with a stronger signal reported in APOE epsilon-4 carriers [66]. This remains a hypothesis: confirmatory randomized trials have not established cognitive benefit, subgroup findings require prospective validation, and the corresponding vitamin D question has been tested and was null [63,65].

Baseline nutritional status as a determinant of triad response: The same logic extends to the full triad. The response to vitamin D concentrates in individuals who are deficient at baseline, whereas supplementation of replete populations yields little [19,64,74]; the scope for vitamin K2 to increase MGP and osteocalcin carboxylation depends on pre-existing undercarboxylation, that is, on vitamin K insufficiency [21,22]. Accordingly, the plausibility of benefit from the combined regimen is greatest in individuals with demonstrable insufficiency and/or elevated risk, and least in replete, low-risk populations—an argument for risk- and biomarker-stratified evaluation in future trials rather than for indiscriminate supplementation. This remains an argument about where benefit is plausible, not a demonstration that benefit exists.

## 8. Beyond Supplementation: Lifestyle and Psychosocial Context

The heart–brain–bone axis should not be construed as governed solely by nutrient intake. The very nodes on which the triad is proposed to act—inflammation, endothelial function, bone remodeling, and cognitive resilience—are also powerfully shaped by modifiable lifestyle and psychosocial factors, which therefore constitute both genuine effect modifiers and potential confounders of supplementation studies.

Physical activity exerts anti-inflammatory effects, improves endothelial function, and provides the mechanical loading that drives bone formation, consistent with the recognition of bone and muscle as endocrine organs engaged in inter-organ communication [6]. Adequate sleep and circadian regularity influence metabolic and inflammatory tone, while chronic psychological stress and social isolation are associated with sustained hypothalamic–pituitary–adrenal and sympathetic activation that can promote the same NF-kappaB-driven inflammatory and catabolic pathways implicated in inflammaging [42,43]. Conversely, positive affect and emotional resilience have been associated with a lower inflammatory burden. The magnitude of these lifestyle and psychosocial influences on the shared nodes of the axis is at least comparable to, and plausibly greater than, that attributable to nutrient supplementation, which is one reason why supplementation trials conducted without measuring them are difficult to interpret.

Two implications follow: Clinically, nutrient supplementation should be viewed as one component of a broader healthy-aging strategy—encompassing regular physical activity, adequate sleep, stress reduction, and social connection—rather than as a stand-alone intervention. Methodologically, lifestyle and psychosocial variables are plausible effect modifiers and confounders that future supplementation trials should measure and, where feasible, use for stratification, lest their omission obscure or exaggerate nutrient effects.

## 9. Safety, Tolerability, and Interactions

Any discussion of combined supplementation is incomplete without an explicit account of harm. The three nutrients are widely regarded as benign, and at customary intakes they generally are, but each carries at least one recognized, evidence-based safety signal, and one carries a drug interaction of genuine clinical consequence. The signals are summarized in Table 7 and discussed below. The evidence base for harm is, if anything, more directly relevant to practice than the evidence base for benefit because it derives from the same large randomized trials but does not depend on the population selection that conditions efficacy.

### 9.1. Long-Chain Omega-3: Atrial Fibrillation and Bleeding

Atrial fibrillation is the best-characterized adverse effect of long-chain omega-3 supplementation. A meta-analysis of seven cardiovascular outcome trials including 81,210 participants found that marine omega-3 supplementation increased the risk of atrial fibrillation (hazard ratio 1.25, 95% confidence interval 1.07–1.46), with a clear dose-dependence: the hazard ratio was 1.49 (1.04–2.15) in trials testing more than 1 g/day and 1.12 (1.03–1.22) in trials testing 1 g/day or less, and meta-regression estimated an 11% increase in risk per additional gram per day [75]. Consistent signals are reported in the trial-level literature and in subsequent meta-analyses [55,56]. This is a quantitatively modest but real hazard that must be weighed against the benefit observed in REDUCE-IT—a trial that itself reported more hospitalizations for atrial fibrillation in the active arm [48]—and it argues against the casual use of doses above 1 g/day outside the indications for which they have been tested. Bleeding risk, by contrast, has been substantially overstated. Long-chain omega-3 prolongs bleeding time in vitro, and modestly increases reported bleeding events without an excess of serious bleeding in REDUCE-IT [48]; a randomized trial of perioperative fish oil in 1516 patients undergoing cardiac surgery found no increase in major perioperative bleeding, and if anything the opposite [76]. Routine discontinuation before surgery is therefore not supported by randomized evidence, although individualized assessment remains appropriate in patients receiving antiplatelet or anticoagulant therapy. Gastrointestinal intolerance and eructation are common but mild and formulation-dependent, and high-dose DHA-containing preparations can raise LDL-cholesterol, which is one of the arguments advanced for EPA-only products [25,56].

### 9.2. Vitamin D3: Excess Intake, Bolus Dosing and Calcium Load

For vitamin D3, the relevant lesson from the randomized literature is that more is not better. Hypercalcaemia, hypercalciuria and frank toxicity are rare at intakes within the tolerable upper intake level of 100 micrograms (4000 IU) per day established by the Institute of Medicine, but they occur predictably at sustained supraphysiological intakes [77,78]. Three randomized findings define the safety envelope. First, large intermittent boluses are harmful: a single annual oral dose of 500,000 IU in 2256 community-dwelling older women increased falls (rate ratio 1.15, 95% CI 1.02–1.30) and fractures (1.26, 1.00–1.59) relative to placebo [79], and monthly high-dose regimens (60,000 IU) increased the incidence of falls compared with a 24,000 IU monthly regimen despite achieving higher 25(OH)D concentrations [80]. Second, high daily doses may be skeletally counterproductive: over three years, 4000 IU/day and 10,000 IU/day produced dose-dependent reductions in radial and tibial volumetric bone mineral density relative to 400 IU/day, with no gain in bone strength [81]. Third, in a replete, unselected population, 2000 IU/day conferred no fracture benefit at all [74]. Taken together, these results argue for daily rather than bolus administration, for intakes within the upper level, and for supplementation targeted at documented insufficiency rather than applied indiscriminately. Because vitamin D3 increases intestinal calcium absorption, particular caution is warranted in patients with granulomatous disease, primary hyperparathyroidism, nephrolithiasis, or advanced chronic kidney disease, in whom monitoring of serum and urinary calcium is appropriate.

### 9.3. Vitamin K2: Interaction with Anticoagulant Therapy

Vitamin K2 is well-tolerated: randomized trials have administered MK-7 at 180–360 micrograms/day for up to three years and 720 micrograms/day for two years without a consistent adverse-event signal [21,24,59], and no tolerable upper intake level has been established for menaquinones. The important issue is not toxicity but interaction. Vitamin K antagonists such as warfarin and acenocoumarol act by depleting reduced vitamin K, and supplemental menaquinone directly opposes them. A dose–response study in anticoagulated volunteers found that MK-7 at intakes as low as 10 micrograms per day—well below the 45 microgram dose common in retail supplements—produced a clinically relevant lowering of the international normalized ratio in a substantial proportion of subjects, and that 45 micrograms per day lowered the international normalized ratio by approximately 40% [82]. MK-7 supplements should therefore be avoided in patients receiving vitamin K antagonist therapy; where their use is unavoidable, the dose must be kept constant, and anticoagulation monitored closely. This interaction is a material constraint on the population in whom combined supplementation could be contemplated, since patients with established cardiovascular disease—precisely those in whom the vascular hypothesis is most attractive—are disproportionately likely to be anticoagulated. Direct oral anticoagulants do not act through gamma-carboxylation and no interaction is expected on mechanistic grounds, but this has not been formally studied. Long-term safety beyond approximately three years of continuous supplementation has not been characterized for any menaquinone.

### 9.4. The Combination and Special Populations

Two points concerning the combination itself deserve emphasis. First, no trial has been designed to assess the safety of the three nutrients administered together, so the safety of the combination cannot be inferred from single-nutrient data; the assumption of simple additivity is untested. Second, the theoretical rationale most often advanced for combining vitamin K2 with vitamin D3—that the former mitigates a vascular hazard of the latter—should not be inverted into a license for higher vitamin D3 intakes. There is no evidence that co-administration of MK-7 renders supraphysiological vitamin D doses safe, and the harms of high-dose vitamin D documented above were observed in trials in which the skeletal and fall outcomes, not vascular calcification, were affected. Combined supplementation should, on the current evidence, be confined to research settings or to clinician-supervised use in individuals with documented insufficiency, with particular attention to anticoagulant therapy, to a history of atrial fibrillation, and to disorders of calcium metabolism.

## 10. Potential Clinical Implications and Research Priorities

Building on the population (Section 7), lifestyle (Section 8) and safety (Section 9) considerations above, this section describes the doses actually used in the studies reviewed and sets out research priorities. Nothing in this section constitutes a therapeutic recommendation, and no combined regimen is proposed.

### 10.1. Doses Used in the Studies Reviewed (Descriptive, Not Recommendations)

It is important to be explicit about what can and cannot be said regarding dose. The optimal dose of each nutrient, the optimal ratio between them, and the optimal duration of combined administration are all unknown, and no combination has been tested against its components. This section therefore describes the doses that were administered in the studies appraised in Section 5 and Section 9, so that readers can see the range over which the evidence was generated; it does not recommend a dose. Across the randomized literature, vitamin D3 was given at 400–10,000 IU/day in daily dosing trials (2000 IU/day in VITAL and DO-HEALTH), and at 60,000 IU monthly or 500,000 IU annually in the bolus trials that reported harm; the tolerable upper intake level is 4000 IU/day, doses above it produced no benefit and measurable skeletal disadvantage, and serum 25(OH)D is the status biomarker used throughout [18,19,64,74,77,79,80,81]. Vitamin K2 as MK-7 was given at 180 micrograms/day in the bone and arterial-stiffness trials, 360 micrograms/day in VitaK-CAC and the diabetes and menopausal trials, and 720 micrograms/day in AVADEC; carboxylation biomarkers (undercarboxylated osteocalcin, dephospho-uncarboxylated MGP) responded across this entire range, whereas clinical and structural endpoints did not, and the highest dose tested was used in the trial with the clearly null structural result [21,24,58,59,61]. Long-chain omega-3 was given at approximately 1 g/day of EPA + DHA in GISSI-Prevenzione, VITAL and ASCEND and at 4 g/day in REDUCE-IT and STRENGTH, with 900 mg/day of DHA in the cognitive trial MIDAS; the Omega-3 index (erythrocyte EPA + DHA content) is the corresponding status biomarker, the only positive hard-endpoint trial used 4 g/day of an EPA ethyl ester in a selected population, and the atrial-fibrillation signal is dose-dependent above 1 g/day [47,48,50,51,52,62,75]. These ranges are summarized in Table 8. They are descriptive of the evidence base, are not individualized medical advice, and should not be read as a recommended regimen; any supplementation, and in particular any vitamin K intake in patients receiving anticoagulants, requires clinician supervision.

### 10.2. Future Perspectives

**Figure 4:**
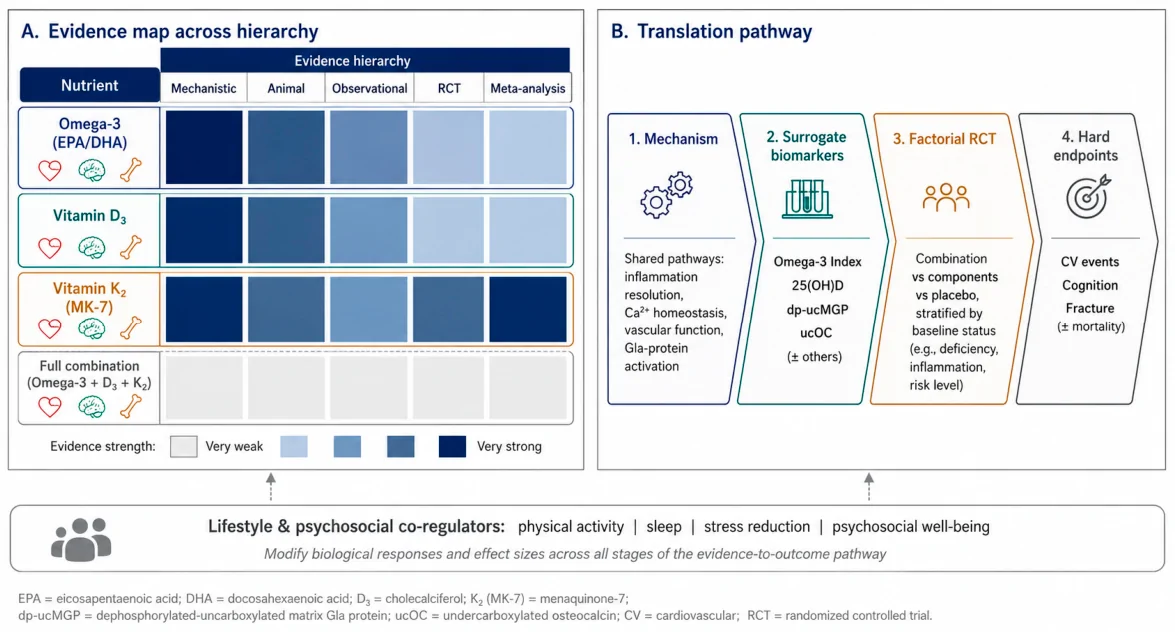
Evidence map and clinical translation framework. (**A**) Evidence map across the evidence hierarchy: for each nutrient (omega-3 (EPA/DHA), vitamin D3, vitamin K2 (MK-7)) and for the full combination of the three, the strength of the current evidence is shown at each level of the hierarchy (mechanistic, animal, observational, randomized controlled trial, meta-analysis). Cell shading encodes the strength of evidence on the five-step scale given in the key beneath the panel, from very weak (pale grey) to very strong (dark navy); the shading reflects the qualitative appraisal defined in Section 2.5 and Table 2 and is not a quantitative measure. The heart, brain, and bone icons beside each nutrient indicate that the appraisal spans all three organ axes, and the row for the full combination is uniformly at the lowest level because no trial has tested the three nutrients together. The panel shows where the evidence is comparatively firm (e.g., MK-7 and bone biomarkers; EPA and high-risk cardiovascular prevention) and where it is preliminary or discordant (e.g., bone–brain endocrine crosstalk; vitamin K2 and structural vascular endpoints; the full triad for multi-organ endpoints). (**B**) Translation pathway: the four sequential steps required to move from mechanism (step 1) through surrogate biomarkers (step 2) and an adequately powered factorial randomized controlled trial (step 3) to hard clinical endpoints (step 4); the chevrons point in the direction of translation, and the color of each chevron identifies the step only and carries no quantitative meaning. The bar beneath both panels shows the lifestyle and psychosocial factors (physical activity, sleep, stress reduction, psychosocial well-being) that act as cross-cutting co-regulators of the axis (Section 8); the dashed arrows denote that these factors modify biological responses and effect sizes at every stage depicted in both panels.

Several priorities emerge. First, and above all, adequately powered factorial-design randomized controlled trials are needed that compare the triad against its individual components and placebo, since no such trial exists; these should enroll appropriately selected (insufficient or higher-risk) populations and stratify by baseline Omega-3 index, 25(OH)D, vitamin K status, and cardiometabolic risk, with prespecified mechanistic biomarkers (Omega-3 index, 25(OH)D, dephospho-uncarboxylated MGP, undercarboxylated osteocalcin) and hard endpoints (cardiovascular events, validated cognitive trajectories, fracture incidence) [56,59]. Second, the dissociation between the carboxylation biomarkers and the clinical endpoints identified in Section 6 should be addressed directly, since a biomarker that responds without predicting outcome is of limited value as a trial endpoint; replication of VitaK-CAC with clinical rather than imaging endpoints is the obvious next step [24,59]. Third, optimal inter-nutrient dosing ratios and the influence of baseline status, genetic polymorphisms (VDR, GGCX, APOE), and the gut microbiome on response must be defined to enable precision-nutrition stratification [41,66]. Fourth, formulation should be standardized and reported (rTG vs. EE; MK-7 vs. MK-4) so that bioavailability does not confound outcome interpretation [15,16]. Fifth, lifestyle and psychosocial co-variables (Section 8) should be measured and, where feasible, used for stratification. Sixth, safety—particularly the omega-3 atrial-fibrillation signal, the harms of high-dose and bolus vitamin D, and the vitamin K-anticoagulant interaction—must be prespecified and rigorously characterized rather than reported as incidental adverse events [75,82]. Figure 4 maps the current evidence landscape and the translational pathway from mechanism to confirmatory trials.

## 11. Conclusions

What is known: The cardiovascular, central nervous, and skeletal systems are mechanistically coupled through shared calcium-handling and inflammatory pathways, a common vascular conduit, and endocrine osteokine signaling; the heart–brain–bone axis is the proposed conceptual framework that assembles these couplings, not a validated physiological entity. Within this framework, vitamin D3 secures calcium supply and exerts VDR-mediated pleiotropy; vitamin K2 (MK-7) activates MGP and osteocalcin in a manner proposed to influence whether calcium is incorporated into bone or deposited in the arterial wall, offering a candidate account of the calcium paradox; and long-chain omega-3 (EPA/DHA) supplies the substrate for specialized pro-resolving mediator biosynthesis and membrane integrity. The comparatively firmer clinical observations are MK-7’s consistent effects on bone and calcification biomarkers, its effect on bone mineral density in one three-year postmenopausal trial and on coronary calcification progression in one two-year imaging trial, and the benefit of high-dose EPA (as an ethyl ester) in selected high-risk cardiovascular patients.

What remains uncertain: The mechanistic case consistently exceeds the clinical evidence. Omega-3 cardiovascular randomized trials are discordant and appear population- and dose-dependent; cognitive randomized trials are largely null or subgroup-dependent; vitamin K2 evidence still rests substantially on surrogate biomarkers, and the two randomized trials of structural vascular endpoints disagree; and bone–brain endocrine signaling is principally preclinical. Most importantly, no adequately powered randomized controlled trial has demonstrated that the combination of long-chain omega-3, vitamin D3, and MK-7 is superior to its individual components or to placebo for any clinical endpoint; no trial has tested the full triad at all, and the two randomized tests of pairwise combinations (AVADEC, DO-HEALTH) were null. Axis-level complementarity therefore remains a hypothesis rather than a demonstrated effect. With respect to formulation, rTG omega-3 has pharmacokinetic (bioavailability) advantages but has not been shown to produce superior clinical outcomes.

Where benefit is most plausible: Based on current evidence, omega-3 benefit is most plausible in selected higher-risk or nutritionally insufficient populations—such as those with hypertriglyceridemia, established cardiovascular risk, low baseline Omega-3 index, or low habitual fish intake—and is limited or absent in replete, low-risk individuals. The same baseline-status dependence applies to vitamin D and, through carboxylation headroom, to vitamin K2. This favors risk- and biomarker-stratified evaluation in future trials over indiscriminate supplementation; it is not a statement of demonstrable benefit in these groups.

Why the axis matters, in context: The heart–brain–bone framework is valuable because it reframes three leading burdens of aging as manifestations of partly shared, potentially modifiable biology and because it generates testable predictions. Crucially, however, lifestyle and psychosocial factors—physical activity, sleep, stress, and social connection—are co-regulators of the same axis, and nutrient supplementation is best understood as one component of a broader healthy-aging strategy rather than a stand-alone intervention. Each nutrient also carries a recognized safety signal (Section 9) that must enter any risk-benefit assessment.

What future trials are required: Definitive evaluation will require adequately powered, factorial-design randomized controlled trials that compare the combination with its components and placebo; enrol appropriately selected (insufficient or higher-risk) populations; stratify by baseline Omega-3 index, 25(OH)D, vitamin K status, cardiometabolic risk, and lifestyle factors; standardize and report formulation; embed prespecified mechanistic biomarkers; and measure hard clinical endpoints over sufficient duration, while prespecifying and monitoring the known safety signals. Until such data exist, the combined regimen is best characterized as a biologically rational, hypothesis-generating strategy for preserving multi-organ homeostasis across the lifespan, and not as an established therapy.

## Figures and Tables

**Table 1:** Search strategy, eligibility criteria, and study-selection procedure.

Element	Specification
Databases	PubMed/MEDLINE; Embase; Cochrane Library (incl. CENTRAL); Web of Science Core Collection; ClinicalTrials.gov (registered/ongoing trials)
Search period	Database inception to 25 June 2026 (original submission); search updated 7 August 2026 for this revision (final search date)
Language	English
Nutrient term block	(“omega-3” OR “n-3 PUFA” OR eicosapentaenoic OR docosahexaenoic OR “fish oil” OR “icosapent ethyl” OR “re-esterified triglyceride”) OR (“vitamin D” OR cholecalciferol OR “25-hydroxyvitamin D” OR calcitriol) OR (“vitamin K” OR menaquinone OR “MK-7” OR “MK-4” OR natto OR phylloquinone)
Organ/outcome term block	(cardiovascular OR “major adverse cardiovascular events” OR “coronary artery calcification” OR “aortic valve calcification” OR “arterial stiffness” OR atherosclerosis) OR (cognition OR dementia OR Alzheimer OR “cognitive decline” OR “neurovascular coupling” OR “blood–brain barrier”) OR (bone OR osteoporosis OR “bone mineral density” OR fracture OR osteocalcin)
Mechanism term block	(“matrix Gla protein” OR “gamma-carboxylation” OR “specialized pro-resolving mediators” OR resolvin OR protectin OR inflammaging OR “vitamin D receptor” OR FGF23 OR osteokine OR GPR158 OR RUNX2 OR RANKL)
Design filters	Randomized controlled trial; meta-analysis; systematic review; cohort studies (applied to the clinical searches only; no design filter for the mechanistic searches)
Supplementary searching	Backward citation tracking of all included trials and reviews; forward citation tracking; hand-search of principal nutrition and cardiovascular journal contents over the final five years
Inclusion criteria	Randomized trials and their systematic reviews/meta-analyses of omega-3, vitamin D3 or vitamin K with cardiovascular, cognitive or skeletal outcomes; prospective cohort or nested case–control studies of intake/status and the same outcomes; mechanistic studies addressing a pathway explicitly linked to the proposed axis; authoritative reviews, consensus statements and dietary reference intake reports
Exclusion criteria	Non-peer-reviewed sources (preprints, abstracts without full report); case reports/series without mechanistic contribution; non-English publications; alpha-linolenic acid only; vitamin K1 studies without bearing on the menaquinone questions (ECKO retained deliberately as a null comparator); animal studies without a counterpart in the human evidence assembled here; duplicate reports of the same trial
Screening and selection	Title/abstract and full-text screening performed independently by two authors (S.-C.F., M.-K.H.); disagreements resolved by discussion, residual disagreements adjudicated by a third author (C.-C.W.)
Data extracted	Design; population and sample size; intervention (molecular form, dose); comparator; duration; prespecified primary outcome; direction of principal result; interpretive limitations (reported in Section 5)
Risk of bias	No formal instrument applied (narrative design); trial-level limitations reported explicitly for every trial in Section 5 and revisited in Section 6

**Table 2:** Evidence-grading framework used in the evidence hierarchy presented in Section 5. Symbols are applied separately within each evidence stream (preclinical, observational, randomized, meta-analytic); the composite “overall strength” categories are anchored to the best available human evidence.

Symbol/Category	Label	Operational Definition
(-)	Absent or insufficient	No directly relevant studies of that design, or only isolated reports insufficient to support a directional inference.
(+)	Limited	A single study, or several small or heterogeneous studies; findings inconsistent, restricted to surrogate endpoints, or at high risk of bias or confounding.
(++)	Moderate	At least two independent studies of adequate design and size with a broadly consistent direction of effect; or one adequately powered randomized trial reporting a surrogate or imaging endpoint.
(+++)	Strong/consistent	Multiple independent, adequately powered studies (or meta-analyses of such studies) with a consistent direction of effect and no major unexplained discordance within that evidence stream.
Overall: Hypothesis-generating	Composite category	Mechanistic and/or observational support only; no randomized evidence for the relationship as stated.
Overall: Limited	Composite category	Randomized evidence exists but is largely null, subgroup-dependent, discordant, or confined to small trials.
Overall: Moderate	Composite category	Consistent randomized evidence for surrogate endpoints, or randomized evidence for clinical endpoints that is discordant but explicable by population or dose.
Overall: Moderate-Strong	Composite category	Consistent randomized evidence across more than one trial for surrogate and/or intermediate endpoints, supported by meta-analysis.
Overall: Strong	Composite category	Consistent randomized evidence for hard clinical endpoints replicated across trials. No relationship appraised in this review reached this category.

Note: “-“: insufficient evidence; “+”: limited evidence; “++”: moderate evidence; “+++”: strong evidence.

**Table 3:** Comparative biochemical and pharmacokinetic properties of the three lipid-soluble nutrients.

Property	Omega-3 (EPA/DHA)	Vitamin D3	Vitamin K2 (MK-7)
Molecular class	Long-chain n-3 PUFA (TG backbone)	Secosteroid pro-hormone	Long-chain menaquinone
Active/effector species	EPA, DHA → SPMs (resolvins, protectins)	1,25(OH)2D (calcitriol)	Reduced MK-7 (GGCX cofactor)
Primary receptor/target	GPR120/FFAR4; membrane incorporation	Nuclear VDR-RXR heterodimer	gamma-Glutamyl carboxylase (GGCX)
Key downstream proteins	Pro-resolving mediator cascade	Calbindin, TRPV6; osteocalcin (expr.)	Carboxylated MGP and osteocalcin
Bioavailability note	rTG > natural TG > EE absorption	Enhanced by dietary-lipid co-ingestion	MK-7 half-life (days) >> MK-4 (hours)
Axis relevance	Anti-inflammatory; Heart + Brain	Calcium uptake; all three organs	Proposed calcium routing (hypothesis); Heart + Bone

**Table 4:** Mechanistic integration of the proposed heart–brain–bone axis across the three nutrients. Entries summarize the principal proposed mechanism in each organ and are mechanistic propositions, not demonstrated clinical effects; the strength of supporting evidence is graded in Section 5.

Pathway/Shared Node	Heart/Vasculature	Brain	Bone	Omega-3 (EPA/DHA)	Vitamin D3	Vitamin K2 (MK-7)
Calcium handling	MGP-gated arterial calcification	Cerebral microvascular calcification; Ca^2+^ excitotoxicity	Calcium uptake and matrix mineralization	Indirect	Increases Ca^2+^ supply (TRPV6/calbindin)	Carboxylates MGP and osteocalcin; proposed influence on site of Ca^2+^ deposition
Inflammation resolution	Resolves endothelial inflammation	Resolves microglial neuroinflammation	Lowers RANKL-driven resorption	SPMs (resolvins, NPD1)	VDR anti-inflammatory transcription	Reported anti-inflammatory effects
Endothelial/microvascular function	Preserves endothelium; lowers TG	Maintains NVU and BBB integrity	Supports marrow microcirculation	Improves endothelial function	VDR endothelial signaling	Proposed inhibition of microvascular calcification
Gla-protein carboxylation	Active MGP	Gas6-TAM neuronal survival	Active osteocalcin	None	Upregulates osteocalcin expression	Essential GGCX cofactor
Membrane/endocrine signaling	FGF23-cardiac remodeling axis	DHA membranes; osteocalcin-GPR158	Osteokine secretion	DHA membrane incorporation	Calcitriol genomic signaling	Activates osteocalcin as osteokine

**Table 5:** Evidence hierarchy for the principal nutrient–organ relationships. Symbols are defined in Table 2 (Section 2.5): (-) absent/insufficient, (+) limited, (++) moderate, (+++) strong/consistent, applied within each evidence stream. Overall strength is anchored to the best available human evidence.

Topic	Preclinical	Observational	RCT	Meta-Analysis	Overall Strength
Omega-3 and CV events	+++	++	++ (discordant)	++	Moderate (population/dose-dependent)
Omega-3 and cognition	+++	++	+ (mostly null)	+	Limited
Vitamin D and bone/calcium	+++	++	++	++	Moderate
Vitamin D and cognition	++	++	+ (null)	+ (obs.)	Limited (baseline-dependent)
Vitamin K2 (MK-7) and bone	+++	++	++	++	Moderate-Strong (surrogate/BMD)
Vitamin K2 and vascular calcification	+++	++	++/+ (discordant imaging endpoints)	+	Limited-Moderate (discordant imaging endpoints)
Heart–Brain–Bone integration	++	+	- (triad untested)	-	Hypothesis-generating

Note: a grade of (+++) in the Preclinical column denotes reproducible mechanistic demonstration across independent models and does not imply clinical proof. No relationship in this review reached the “Strong” overall category, which requires consistent randomized evidence for hard clinical endpoints replicated across trials.

**Table 6:** Major clinical trials and analyses relevant to the triad, with the direction of the principal result. EE, ethyl ester; IPE, icosapent ethyl; CAC, coronary artery calcification; AVC, aortic valve calcification; MACE, major adverse cardiovascular events; BMD, bone mineral density; dp-ucMGP, dephospho-uncarboxylated matrix Gla protein.

Study (Design)	Population (*n*)	Intervention	Duration	Primary Outcome	Direction	Key Findings	Limitations
GISSI-P (RCT)	Post-MI (11,324)	n-3 PUFA ~1 g/d	3.5 y	Death/CV events	Positive	Reduced events and mortality	Open-label; pre-statin era
REDUCE-IT (RCT)	Statin-treated, high TG (8179)	IPE 4 g/d (EPA-EE)	~4.9 y	MACE composite	Positive	~25% MACE reduction; more AF hospitalizations	Mineral-oil placebo debate; EPA-only; not an rTG preparation
EVAPORATE (RCT)	High TG on statin (80)	IPE 4 g/d	18 mo	Plaque volume (CT)	Positive	Low-attenuation plaque regression	Small; imaging surrogate
STRENGTH (RCT)	High CV risk (13,078)	EPA + DHA 4 g/d (carboxylic acid) vs. corn oil	~3.5 y	MACE composite	Null	No benefit; increased AF	Contrasts REDUCE-IT; formulation and comparator differ
VITAL (RCT)	Primary prevention (25,871)	EPA + DHA 1 g/d and/or vitamin D3 2000 IU/d	~5.3 y	MACE and cancer	Null	No significant CV benefit	Low dose; healthy, largely replete cohort
ASCEND (RCT)	Diabetes (15,480)	n-3 1 g/d	~7.4 y	Vascular events	Null	No benefit	Low dose
VITAL fracture ancillary (RCT)	Midlife/older adults (25,871)	Vitamin D3 2000 IU/d	~5.3 y	Incident fracture	Null	No effect on total, non-vertebral, or hip fracture	Not selected for deficiency or low bone mass
MIDAS (RCT)	Age-related cognitive decline (485)	DHA 900 mg/d	24 wk	Memory tests	Positive	Improved episodic and visuospatial memory	Short; surrogate cognitive endpoints
MAPT (RCT)	Memory complaints, elderly (1680)	Omega-3 ± multidomain intervention	3 y	Cognitive composite	Null	No overall benefit; subgroup signal only	Low baseline omega-3 deficiency; subgroup finding unvalidated
DO-HEALTH (RCT, factorial)	Healthy seniors (2157)	Vitamin D3 + omega-3 + exercise	3 y	Composite clinical	Null	No effect on prespecified primary outcomes	Replete, healthy cohort; only randomized test of a D3 + omega-3 pairing
VITAL cognitive ancillary (RCT)	Adults ≥ 60 y (3424/794)	Vitamin D3 2000 IU/d	2.0–2.8 y	Global cognitive composite	Null	No effect on rate of decline; no interaction with baseline 25(OH)D	Ancillary; modest duration
Knapen (RCT)	Postmenopausal women (244)	MK-7 180 mcg/d	3 y	BMD and osteocalcin carboxylation	Positive	Reduced BMD loss; improved carboxylation	Surrogate/BMD, not fracture
Knapen arterial stiffness (RCT)	Healthy postmenopausal women (244)	MK-7 180 mcg/d	3 y	Arterial stiffness indices	Positive	Reduced carotid-femoral pulse wave velocity and stiffness index; dp-ucMGP −50%	Intermediate endpoint; effect largest in stiffest subgroup
Emaus (RCT)	Early menopausal women (334)	MK-7 (natto-derived) 360 mcg/d	12 mo	Bone loss rate	Null	ucOC reduced; no effect on bone loss at any site	Twelve months may be short for BMD
ECKO (RCT)	Postmenopausal osteopenia (440)	Vitamin K1 5 mg/d	2–4 y	BMD	Null	No protection against BMD decline; fewer fractures/cancers in exploratory analyses	Phylloquinone, not menaquinone; secondary findings exploratory
VitaK-CAC (RCT)	Coronary artery disease (180)	MK-7 360 mcg/d	2 y	CAC progression	Positive	Significantly less CAC progression than placebo	Imaging endpoint; single trial; needs replication
AVADEC (RCT)	Elderly men with AVC score >300 AU (365)	MK-7 720 mcg/d + vitamin D 25 mcg/d	2 y	AVC progression	Null	No effect on AVC or coronary calcification progression, valve surgery, death, or CV events, despite large dp-ucMGP reduction	Men only; advanced calcification at baseline
AVADEC substudy (RCT)	Aortic valve calcification	Vitamin K2 + D3	24 mo	Epicardial fat/inflammation	Null	K-status marker changed; no effect on epicardial adipose tissue or inflammation	Substudy; surrogate endpoints
Zwakenberg (RCT)	Type 2 diabetes with vascular calcification (68)	MK-7 360 mcg/d	6 mo	Arterial calcification (CT/18F-NaF PET)	Null	dp-ucMGP markedly reduced; no reduction in calcification on CT	Small; short; surrogate imaging

**Table 7:** Recognized safety signals, tolerability considerations and interactions for the three nutrients, with the practical implication of each. AF, atrial fibrillation; INR, international normalized ratio; UL, tolerable upper intake level; VKA, vitamin K antagonist; BMD, bone mineral density.

Nutrient	Signal or Interaction	Best Available Evidence	Direction and Magnitude	Practical Implication
Omega-3 (EPA/DHA)	Atrial fibrillation	Meta-analysis of 7 cardiovascular outcome RCTs (81,210 participants)	Increased risk: HR 1.25 (95% CI 1.07–1.46); HR 1.49 for >1 g/d vs. 1.12 for ≤1 g/d; +11% per additional g/d	Avoid doses >1 g/d outside tested indications; particular caution with prior AF or AF risk factors
Omega-3 (EPA/DHA)	Bleeding	REDUCE-IT; randomized perioperative trial (1516 cardiac surgery patients)	Modest increase in reported bleeding without excess serious bleeding; no increase in major perioperative bleeding	Routine pre-operative discontinuation is not supported; individualize where antithrombotics are used
Omega-3 (EPA/DHA)	GI intolerance; LDL-C rise with DHA-containing preparations	Randomized trials and reviews	Common but mild; LDL-C effect is formulation-dependent	Dose titration; lipid monitoring at high doses
Vitamin D3	Hypercalcaemia, hypercalciuria, toxicity	Clinical reviews; Institute of Medicine dietary reference intake report	Rare within the UL of 100 mcg (4000 IU)/d; predictable at sustained supraphysiological intakes	Do not exceed the UL without monitoring; check 25(OH)D and calcium at high doses
Vitamin D3	Large intermittent (bolus) dosing	RCT: 500,000 IU annually (2256 women); RCT: 60,000 IU monthly (200 adults)	Increased falls (RR 1.15) and fractures (RR 1.26); increased incidence of falls	Bolus regimens should be avoided; use daily dosing
Vitamin D3	High daily dose and bone density	RCT: 400 vs. 4000 vs. 10,000 IU/d over 3 y	Dose-dependent reduction in radial and tibial volumetric BMD; no gain in bone strength	Higher is not better; no skeletal rationale for doses above the UL
Vitamin D3	Absence of benefit in replete populations	VITAL fracture ancillary study (25,871)	No reduction in total, non-vertebral or hip fracture	Target documented insufficiency rather than supplement indiscriminately
Vitamin K2 (MK-7)	Vitamin K antagonist interaction	Dose–response RCT in VKA-treated volunteers	MK-7 from 10 mcg/d produced clinically relevant INR lowering in a substantial proportion; 45 mcg/d lowered INR by ~40%	Avoid MK-7 supplements during VKA therapy; if unavoidable, fix the dose and monitor INR closely
Vitamin K2 (MK-7)	Direct oral anticoagulants	No mechanistic basis for interaction (DOACs act independently of gamma-carboxylation); not formally studied	No interaction expected	No specific restriction identified; clinical confirmation lacking
Vitamin K2 (MK-7)	General tolerability	RCTs at 180–360 mcg/d for up to 3 y; 720 mcg/d for 2 y	No consistent adverse-event signal; no UL established	Long-term safety beyond ~3 years is not characterized
Combination	Triad administered together	No dedicated safety study	Not characterized	Safety of the combination cannot be inferred from single-nutrient data

**Table 8:** Doses administered in the clinical studies appraised in this review by nutrient. The table is descriptive of the published evidence base and is not a dosing recommendation; the optimal dose of each nutrient and the optimal ratio for combined use are unknown.

Nutrient	Doses Administered in the Studies Reviewed	Representative Studies	Status Biomarker Used	Comment
Vitamin D3 (cholecalciferol)	400–10,000 IU/d in daily dosing trials (2000 IU/d in VITAL and DO-HEALTH; ~800–2000 IU/d in most musculoskeletal maintenance studies); 60,000 IU monthly and 500,000 IU annually in bolus trials	VITAL [50]; DO-HEALTH [64]; Burt [81]; Sanders [79]; Bischoff-Ferrari [80]	Serum 25(OH)D	UL is 4000 IU/d; doses above it gave no benefit and lower volumetric BMD, and bolus regimens increased falls and fractures
Vitamin K2 (MK-7)	180 mcg/d (bone and arterial-stiffness trials); 360 mcg/d (VitaK-CAC, diabetes calcification trial, early-menopausal bone trial); 720 mcg/d (AVADEC); 10–45 mcg/d in the anticoagulation dose–response study	Knapen 2013 [21]; Knapen 2015 [58]; VitaK-CAC [59]; Zwakenberg [61]; Emaus [72]; AVADEC [24]; Theuwissen [82]	ucOC; dp-ucMGP	Biomarkers responded across the whole range; structural endpoints did not, and the highest dose was used in the trial with the null structural result
Omega-3 (EPA/DHA)	~1 g/d EPA + DHA (GISSI-Prevenzione, VITAL, ASCEND); 4 g/d (REDUCE-IT as EPA ethyl ester; STRENGTH as carboxylic acid); 900 mg/d DHA (MIDAS)	GISSI-P [47]; VITAL [50]; ASCEND [51]; REDUCE-IT [48]; STRENGTH [52]; MIDAS [62]	Omega-3 index (erythrocyte EPA + DHA %)	The only positive hard-endpoint trial used 4 g/d of an EPA ethyl ester in a selected population; the AF signal is dose-dependent above 1 g/d
The three nutrients combined	No study has administered all three together	None	-	No combined dose can be derived from the evidence reviewed here

Note: no study has administered all three nutrients together, so no combined dose can be derived from the evidence reviewed here. IU, international units; UL, tolerable upper intake level; dp-ucMGP, dephospho-uncarboxylated matrix Gla protein; ucOC, undercarboxylated osteocalcin.

## Data Availability

No new data were created or analyzed in this study. Data sharing is not applicable to this article.

## Abbreviations

The following abbreviations are used in this manuscript:

1,25(OH)_2_D	1,25-dihydroxyvitamin D (calcitriol)
25(OH)D	25-hydroxyvitamin D
AF	atrial fibrillation
APOE	Apolipoprotein E
AVC	Aortic valve calcification
BBB	Blood–brain barrier
BMD	Bone mineral density
BMP-2	Bone morphogenetic protein-2
CAC	Coronary artery calcification
CENTRAL	Cochrane Central Register of Controlled Trials
CI	Confidence interval
cMGP	Carboxylated matrix Gla protein
CVD	Cardiovascular disease
CYP27B1	Cytochrome P450 27B1 (25-hydroxyvitamin D 1α-hydroxylase)
CYP2R1	Cytochrome P450 2R1 (vitamin D 25-hydroxylase)
DHA	Docosahexaenoic acid
DOAC	Direct oral anticoagulant
dp-ucMGP	Dephospho-uncarboxylated matrix Gla protein
EE	Ethyl ester
EPA	Eicosapentaenoic acid
FFAR4	Free fatty acid receptor 4 (GPR120)
FGF23	Fibroblast growth factor 23
Gas6	Growth arrest-specific protein 6
GGCX	γ-glutamyl carboxylase
GPR158	G protein-coupled receptor 158
GRADE	Grading of Recommendations, Assessment, Development and Evaluations
HPA	Hypothalamic–pituitary–adrenal (axis)
HR	Hazard ratio
ICAM-1	Intercellular adhesion molecule-1
IL-6	Interleukin-6
INR	International normalized ratio
IPE	Icosapent ethyl
MACE	Major adverse cardiovascular events
MGP	Matrix Gla protein
MK-4	Menaquinone-4
MK-7	Menaquinone-7
n-3 PUFA	Omega-3 polyunsaturated fatty acid
NF-κB	Nuclear factor kappa B
NPD1	Neuroprotectin D1
OPG	Osteoprotegerin
PRISMA	Preferred Reporting Items for Systematic Reviews and Meta-Analyses
RANKL	Receptor activator of NF-κB ligand
RCT	Randomized controlled trial
rTG	Re-esterified triglyceride
RUNX2	Runt-related transcription factor 2
RXR	Retinoid X receptor
SPM	Specialized pro-resolving mediator
TAM	Tyro3/Axl/MerTK receptor tyrosine kinase family
TG	Triglyceride
TNF-α	Tumor necrosis factor-alpha
TRPV6	Transient receptor potential vanilloid 6
ucMGP	Undercarboxylated matrix Gla protein
ucOC	Undercarboxylated osteocalcin
UL	Tolerable upper intake level
VCAM-1	Vascular cell adhesion molecule-1
VDR	Vitamin D receptor
VDRE	Vitamin D response element
VKA	Vitamin K antagonist
